# Deeper, longer phenotyping to accelerate the discovery of the genetic architectures of diseases

**DOI:** 10.1186/gb4175

**Published:** 2014-05-20

**Authors:** Isaac S Kohane

**Affiliations:** 1 Center for Biomedical Informatics, Harvard Medical School, Boston, MA 01115, USA

## 

A recent National Academy of Sciences report entitled ‘Precision Medicine’ [1] made the point that, in this era of commodity-priced genome-scale measurements, we can now envisage a systematic reclassification of human pathobiology on a population scale. These high-throughput measurement modalities promise greater precision and accuracy to provide patients with individualized diagnoses and therapies. Indeed, we have already seen remarkable success in this regard in improved prognostics and therapeutics for breast cancer [2], non-small-cell lung carcinomas [3] and the leukemias [4] through molecular-based subtype profiling. By contrast, many have written about the artificiality of current organ-based phenotypes and often clinical-department-based diagnoses [5,6] that do not correspond to the underlying pathotypes that cross conventional clinical categorizations. This inadequacy of the current and often-arbitrary clinical classifications, coupled with encouraging results from molecular medicine, has led to a swing of the pendulum to the opposite extreme of where it was in the pre-genomic era. Genotypic variation is often but a small slice of relevant pathotypic variation [7], and the recent call for a sequencing-first approach [8] for molecular-driven classification could result in expensive and frustrating delays in discovering the true genetic architecture of much of human disease. In many cases, taking a more detailed data-driven look at the clinical characterization of individual patients, particularly as revealed by their distinct trajectories over time, might rescue a large number of otherwise-misdirected genomic investigations.

Premature categorization of a clinical phenotype in a genomic case–control study, particularly in complex disease, can lead to an injudicious investment of limited resources for a restricted scientific payoff. First, for example, consider a reasonably common disease, such as autism, affecting over 1% of individuals. Suppose that, like many common diseases, it is suspected that its inherited component is caused by a large set of genetic sequence variants in different genes and even different pathways [9-13]. If each of the disease-causing variants is even modestly rare, then a simple case-controlled study will require numbers of patients orders of magnitude higher than the investigators might be able to recruit. For example, if the disease prevalence is as high as 1%, variant frequency of 1%, relative risk of 2.0, then, with 80% power, discovering each of these variants would require 23,000 subjects [14], which will typically take many years and cost millions of dollars. This imposes a delay to the time when we can better understand the genetic architecture of the disease. Second, it presents a significant economic burden in times of difficult funding for science. This problem is accentuated by the inevitable contributions of noise and bias of environmental exposures to the phenotypic variance as well as the gene-environment interactions [15].

However, with a phenotypic-driven longitudinal approach, researchers will observe individuals who develop clinical findings that are not the primary disease phenotype but are instrumental in understanding the pathobiology of the patient. If the additional clinical findings (for example, co-morbidities) are themselves uncommon (for example, found in 2% or less of the individuals with the primary disease phenotype), even the clinicians caring for the patients might not recognize that there exist groups of patients with archetypal clusters of these co-morbidities. There will therefore be subpopulations of clustered clinical pathologies that would be completely opaque to the original classic approach for a genomic association study. A new and potentially more powerful paradigm for genomic association would include identification of the genetic architectures behind each phenotypic cluster. If there are, for example, 10 such (similarly sized) phenotypic clusters, the frequency of variants that contribute to phenotypes of individual clusters can increase and be as large as 10%. Similarly, they can drop to 0% for those clusters that they do not contribute to. In that case, with a relative risk of 2.0, only 2,300 subjects would have to be studied rather than 23,000 - a change that might make the difference between a successful or a disappointing study. Of course, there are several limiting assumptions implicit in this scenario, including first that the individual genetic variants are contributing to the frequency of co-morbidities in each cluster and, second, that the contributions of the individual variants are identical for each of these co-morbidities.

Despite the aforementioned caveats, we have many examples where better phenotyping enables better understanding of the genetics of disease. For example, whereas 100 years ago heart failure was viewed as a monolithic disease, careful current phenotyping and population studies have revealed heart failure in middle-aged individuals who are highly enriched for the cardiomyopathy gene variants. Similarly, older individuals suffering heart failure due to atherosclerosis have a different set of variants that contribute to the disease. After the fact, it would seem ludicrous to perform a case–control study across all heart failure patients - but, in effect, that is how many of our current and planned studies are structured, although there are notable exceptions (for example, in diabetes [16] and asthma [17]).

As a research community, we can now break free from our definitions of disease and allow the full biological impact of the genetic variants to be expressed across time and across multiple symptom complexes. An important and previously definitive objection to this approach was simply one of cost. Whereas the cost of a whole-genome variant scan is $100 or less and a whole-genome sequence is $1,000, characterizing a patient fully and repeatedly over their lifetime can and will cost many tens of thousands of dollars. Fortunately, as a by-product of the automation of healthcare, there are increasingly large volumes of data that are available across years and decades of a patient’s lifetime over which thousands of different clinical variables are measured [18,19]. Clinical narrative notes from the electronic health record can also be turned into codified variables through the process of natural language processing [20,21]. This now allows the identification of clusters of patients arising over time at a marginal cost of cents per patient and at very high speed. For example, in a recent study of children with autism, it was possible to identify clusters of children with autism and 80% prevalence of seizures, another subgroup with a high prevalence of viral and bacterial infections, and autoimmune diseases, and a third group with a variety of neuropsychiatric diseases such as schizophrenia, attention deficit hyperactivity disorder and anxiety disorders [22]. Therefore, rather than a monolithic disease, autism begins to look more like a set of clinical syndromes that each merits its own independent genetic study, just like the distinct causes of heart failure.

It will become clearer over time that, in this instance, we can have our cake and eat it too. A deeper and longer phenotyping of human populations is more possible than ever before with emerging big-data sets, such as access to biorepositories and longitudinal troves of real-time health information on patients. Just as predicted in the precision-medicine report [1], we can create at minimal incremental cost an ‘information commons’ of a large, even national, population that resolves to the single individual the full array of molecular, genome-scale characterizations. Furthermore, this will permit a deep characterization of the clinical evolution of each of these patients over time so that, in a genuinely data-driven fashion, we can determine what are the true or natural biologically coherent subclasses, whether driven by genetic or environmental influences.

## Competing interests

The author declares that he has no competing interests.
